# Choosing Wisely for Thyroid Conditions: Recommendations of the Thyroid Department of the Brazilian Society of Endocrinology and Metabolism

**DOI:** 10.20945/2359-3997000000323

**Published:** 2021-02-15

**Authors:** Jose Miguel Dora, Rosa Paula Mello Biscolla, Gustavo Caldas, Janete Cerutti, Hans Graf, Ana O. Hoff, Glaucia M. F. S. Mazeto, Patrícia Kunzle Ribeiro Magalhães, Cleo Otaviano Mesa, Rafael Selbach Scheffel, Patricia de Fatima dos Santos Teixeira, Fernanda Vaisman, Danilo Villagelin, Ana Luiza Maia

**Affiliations:** 1 Universidade Federal do Rio Grande do Sul Faculdade de Medicina Hospital de Clínicas de Porto Alegre Porto Alegre RS Brasil Unidade de Tireoide do Hospital de Clínicas de Porto Alegre e Faculdade de Medicina da Universidade Federal do Rio Grande do Sul (UFRGS), Porto Alegre, RS, Brasil; 2 Universidade Federal de São Paulo São Paulo SP Brasil Universidade Federal de São Paulo (Unifesp), São Paulo, SP, Brasil; 3 Universidade Federal de Pernambuco Faculdade de Medicina Recife PE Brasil Faculdade de Medicina da Universidade Federal de Pernambuco (UFPE), Recife, PE, Brasil; 4 Universidade Federal do Paraná Faculdade de Medicina Curitiba PR Brasil Faculdade de Medicina da Universidade Federal do Paraná (UFPR), Curitiba, PR, Brasil; 5 Instituto do Câncer do Estado de São Paulo São Paulo SP Brasil Instituto do Câncer do Estado de São Paulo (ICESP), São Paulo, SP, Brasil; 6 Universidade Estadual Paulista Faculdade de Medicina Botucatu SP Brasil Faculdade de Medicina da Universidade Estadual Paulista (Unesp), Botucatu, SP, Brasil; 7 Universidade de São Paulo Ribeirão Preto SP Brasil Universidade de São Paulo (USP), Ribeirão Preto, SP, Brasil; 8 Universidade Federal do Rio de Janeiro Rio de Janeiro RJ Brasil Universidade Federal do Rio de Janeiro (UFRJ), Rio de Janeiro, RJ, Brasil; 9 Instituto Nacional de Câncer Rio de Janeiro RJ Brasil Instituto Nacional de Câncer (INCA), Rio de Janeiro, RJ, Brasil; 10 Pontifícia Universidade Católica de Campinas Faculdade de Medicina Campinas SP Brasil Faculdade de Medicina da Pontifícia Universidade Católica de Campinas, Campinas, SP, Brasil

**Keywords:** Choosing Wisely, overuse, thyroid, endocrinology

## Abstract

**Objective:**

Choosing Wisely (CW) is an initiative that aims to advance the dialogue between physicians and patients about low-value health interventions. Given that thyroid conditions are frequent in clinical practice, we aimed to develop an evidence-based list of thyroid CW recommendations.

**Materials and methods:**

The Thyroid Department of the Brazilian Society of Endocrinology and Metabolism (SBEM) named a Task Force to conduct the initiative. The Task Force work was based on an electronic Delphi approach. The 10 recommendations that received the highest scores by the Task Force were submitted for voting by all SBEM associates. The 5 recommendations that received the highest scores by SBEM associates are presented herein.

**Results:**

The Task Force was composed of 14 thyroidologists from 10 tertiary-care, teaching-based Brazilian institutions. The brainstorming/ideation phase resulted in 69 recommendations. After the removal of duplicates and recommendations that did not adhere to the initiative's scope, 35 remained. Then the Task Force voted to attribute a grade (0 [lowest agreement] to 10 [highest agreement]) for each recommendation. The 10 recommendations that received the highest scores by the Task Force were submitted to all SBEM associates. A total of 683 associates voted electronically, attributing a grade (0 to 10) for each recommendation. The 5 recommendations that received the highest scores by the SBEM associates compose our final list.

**Conclusion:**

A set of recommendations to avoid unnecessary medical tests, treatments, or procedures for thyroid conditions are offered with a transparent methodology. This initiative aims to foster productive interactions between physicians and patients, stimulating shared decision-making.

## INTRODUCTION

The overuse of low-value interventions is a global problem. A growing body of knowledge highlights that it leads to a waste of precious health care resources and poses patients at risk of harm (1).

Choosing Wisely is a worldwide health care professional-led initiative that aims to advance the dialogue between physicians and patients about low-value health interventions, avoiding wasteful or unnecessary medical tests, treatments, and procedures (2,3).

The Choosing Wisely initiative started in the United States in 2012 led by the American Board of Internal Medicine (ABIM) and is now present in many countries, involving more than one hundred medical associations/societies, hospitals, and universities.

Given that thyroid conditions are widespread and that low-value interventions for these conditions are frequent in clinical practice, we aimed to develop a list of recommendations of measures not to be done when approaching patients with thyroid conditions.

This article aims to summarize the top five recommendations in the list developed by the Thyroid Department of the Brazilian Society of Endocrinology and Metabolism (SBEM) for the Choosing Wisely project.

## MATERIALS AND METHODS

In 2017 the Thyroid Department of the Brazilian Society of Endocrinology and Metabolism (SBEM) and the Choosing Wisely Brasil agreed to work towards the development of a list of Choosing Wisely recommendations for thyroid conditions.

The Thyroid Department named a Chair (JMD) and a Co-chair (ALM) for the initiative. Additional members of SBEM, representing thyroid groups from 10 tertiary care, teaching-based Brazilian institutions, were invited to compose the Task Force. Of note, all members of the Task Force were clinical thyroid specialists, not surgeons.

The Task Force work consisted of 3 phases:

1. brainstorming and ideation was conducted electronically through a Delphi approach in which all Task Force members were invited to submit proposals of recommendations;
2. all proposals of recommendations were reviewed, and duplicates removed;
3. the Task Force graded each recommendation on a 0 (lowest agreement) to 10 (highest agreement) scale.

The 10 recommendations that received the highest scores by the Task Force were submitted for voting by e-mail to all SBEM associates, who graded each recommendation on the 0 (lowest agreement) to 10 (highest agreement) scale.

A final list of the 5 recommendations that received the highest scores by the SBEM associates composes the Choosing Wisely – Thyroid SBEM recommendations.

## RESULTS

The Task Force was composed of 14 thyroidologists from 10 different Brazilian institutions. The brainstorming and ideation phase resulted in 69 recommendations, thus each member of the Task Force contributed with a median of 3 (min.-max. 1-11) recommendations. After the removal of duplicates and recommendations that did not adhere to the scope of the initiative, 35 recommendations remained. Then the 14 members of the Task Force graded each recommendation. The mean grades for the 35 recommendations ranged from 9.4 ± 1.2 to 6.0 ± 2.3. The 10 recommendations that received the highest scores by the Task Force were then submitted to all SBEM associates by e-mail (Table 1). A total of 683 associates graded each recommendation electronically, attributing a grade (0 to 10) for each recommendation. The grades for the 10 recommendations ranged from 9.8 ± 1.2 to 7.6 ± 2.7, and the 5 recommendations that received the highest scores by the SBEM associates compose our final list of recommendations (Table 2).

**Table 1:** Top 10 recommendations of the Thyroid Department of the Brazilian Society of Endocrinology and Metabolism (SBEM) Task Force

Recommendations of the Task Force	Task Force Grades (n = 14)
Do not repeat autoantibodies (antithyroperoxidase [anti-TPO] and antithyroglobulin) measurements in the follow-up of patients with Hashimoto's hypothyroidism who have a previous positive antibody	9.4 ± 1.2
Do not order total T3 and/or free T3 measurements in patients without clinical suspicion or diagnosis of hyperthyroidism/thyrotoxicosis	9.1 ± 1.6
Do not order thyroid ultrasound for nodule detection in patients without thyroid anatomic anomalies on clinical examination*	9.1 ± 1.2
Do not prescribe triiodothyronine (T3), alone or combined with levothyroxine (T4), for hypothyroidism treatment	8.6 ± 1.8
Do not order reverse T3 (rT3) for evaluation of thyroid function	8.9 ± 1.3
Do not order thyroglobulin in the initial evaluation of thyroid nodules	8.9 ± 1.9
Do not perform frequent monitoring (interval less than 6 months) with TSH and free T4 in patients with hypothyroidism who are treated with a stable dose of levothyroxine (LT4) and have no specific clinical indication	8.6 ± 1.8
Do not order molecular markers in the initial evaluation of patients with thyroid nodules	8.6 ± 1.8
Do not administer high radioactive iodine activities for patients with thyroid carcinoma considered to be of low risk	8.3 ± 2.4
Do not maintain suppressive doses of levothyroxine (LT4) in patients with differentiated thyroid carcinoma with excellent response	8.2 ± 2.3

Grades are expressed as mean ± standard deviation.

* This recommendation do not applies for patients with thyroid cancer predisposing conditions such as multiple endocrine neoplasia type 2 (MEN 2) mutations, patients with two or more family members with thyroid carcinoma and for patients with prior radiation of the neck.

**Table 2:** Final Choosing Wisely^®^ recommendations and rationale of the Thyroid Department of the Brazilian Society of Endocrinology and Metabolism (SBEM)

Recommendation	Rationale
Do not order reverse T3 (rT3) for evaluation of thyroid function	Reverse T3 is derived from the inactivation of T4, which occurs predominantly through type 3 deiodinase activity. It is an inactive hormone, and its serum level does not reflect thyroid function. Thus, its measurement has specific indications (most of them in a research setting) and should not be done to evaluate thyroid function (4–7).
Do not prescribe triiodothyronine (T3), alone or combined with levothyroxine (T4), for hypothyroidism treatment	Although the thyroid produces small amounts of T3, there is no evidence that the treatment of hypothyroidism should include T3 seeking improvement of symptoms. T4 is inexpensive, has rapid intestinal absorption and a long half-life (7 days), and allows for single daily intakes and plasma stability of T3 and T4. T4 depends on tissue deiodination for conversion to T3. T3 has a short half-life and requires several daily intakes. Although some experimental animal data suggest that the combination of T4 and T3 may be superior to T4 alone, there is no clear evidence of this effect on humans, so the combination is not routinely recommended (4–6).
Do not repeat autoantibodies (antithyroperoxidase [anti-TPO] and antithyroglobulin) measurements in the follow-up of patients with Hashimoto's hypothyroidism who have a previous positive antibody	Once the autoantibodies (anti-TPO and antithyroglobulin) are positive, the etiology of hypothyroidism is defined, and there is no need to repeat them (4–6).
Do not order thyroglobulin in the initial evaluation of thyroid nodules	Thyroglobulin, a tissue-specific protein, is one of the main parameters in the follow-up of patients with thyroid cancer who have undergone surgery. Serum thyroglobulin levels may increase in various thyroid (benign and malignant) diseases. Thus, serum thyroglobulin measurement does not add information about the nature of thyroid nodules and has no role in thyroid cancer detection (8–10).
Do not order molecular markers in the initial evaluation of patients with thyroid nodules	Fine-needle aspiration biopsy (FNAB) is the most accurate and cost-effective method for evaluating thyroid nodules. Molecular markers should only be used if they improve clinical decision-making. The usefulness of a molecular test should be based on strong evidence that the result will add to the decision-making process, thus justifying its incorporation into clinical practice. It is not the case of molecular markers in the diagnosis of thyroid nodules, which have their nature clarified in about 85% of patients undergoing FNAB. The role of molecular markers may be justified in some cases of nodules in which the FNAB did not provide a definitive diagnosis, respecting the particular context of the patient and the health care setting (8–10).

The Portuguese version of the 5 recommendations is available as Supplementary Material.

## CONCLUSION

In this report, we described the development of the Thyroid Department of the Brazilian Society of Endocrinology and Metabolism (SBEM) Choosing Wisely initiative list.

A set of recommendations to avoid unnecessary medical tests, treatments, or procedures for thyroid conditions is offered with a transparent methodology. This initiative aims to foster productive interactions between physicians and patients, stimulating shared decision-making and elevating the standards of care for thyroid conditions.
